# Supplementary material for: The Time-Dependent Association of Torque Teno Virus Load with the Level of SARS-CoV-2 S1 IgG Antibodies Following COVID-19 Vaccination in Kidney Transplant Recipients
Source: Viruses. 2023 Oct 31;15(11):2189. doi: 10.3390/v15112189 (PMC10674182; doi:10.3390/v15112189)
Supplement: Supplementary file 1 [file viruses-15-02189-s001.zip › viruses-2659697-supplementary.pdf]

Supplementary Table S1. Baseline characteristics of the included and excluded cohort.

	Included cohort<br>(n=94)	Excluded cohort<br>(n=28)
Female, n (%)	41 (43.6)	16 (57.1)
Caucasian, n (%)	91 (96.8)	28 (100)
Age (years)	58.2± 12.2	60.4± 11.7
BMI (kg/m^2)	27.2± 4.7	27.9± 4.3
Comorbidities, n (%)		
- Hypertension	70 (74.5)	21 (75.0)
- Diabetes Mellitus	19 (20.2)	2 (7.1)
- History of malignancy[1]	10 (10.6)	7 (25.0)
- Auto-immune disease	8 (8.5)	1 (3.6)
Lymphocytes (10^9/L)	1.35 (0.9-2.0)	1.24 (1.1-1.9)
eGFR (ml/min/1.73m^2)	50.9±18.9	49.5± 17.4
Primary renal diagnosis, n (%)		
- Immune-mediated disease	11 (11.7)	3 (12.0)
- Interstitial nephritis	7 (7.4)	0 (0.0)
- Familial/hereditary renal diseases	18 (19.1)	4 (16.0)
- Congenital diseases	9 (9.6)	1 (4.0)
- Vascular diseases	8 (8.5)	6 (24.0)
- Diabetic Kidney Disease	6 (6.4)	1 (4.0)
- Other	13 (13.8)	8 (32.0)
- Unknown	22 (23.4)	2 (8.0)
Transplant characteristics		
- First kidney transplant, n (%)	83 (88.3)	21 (75.0)
- Time after last transplantation (months)	41.0 (13.0-85.0)	69.5 (40.8-94.8)*
- Last transplant		
○ Living, n (%)	62 (66.0)	16 (57.1)
○ Pre-emptive, n (%)	39 (41.5)	10 (35.7)
Immunosuppressive treatment, n (%)		
- Steroids	93 (98.9)	28 (100)
- Mycophenolate mofetil	84 (89.4)	28 (100)
- Calcineurin inhibitor	89 (94.7)	26 (92.9)
- Azathioprine	3 (3.2)	0 (0.0)
- mTOR inhibitor	2 (2.1)	0 (0.0)
Number of received COVID-19 vaccinations		
- 0	50 (53.2)	13 (46.34)
- 2	33 (35.1)	12 (42.9)
- 3	11 (11.7)	3 (10.7)

Variables are presented as mean ± SD, or as median (IQ interval) in case of non-normal distribution. P-values are calculated using independent sample t test in case of normal distribution, Mann Whitney U in case of non-normal distribution and Chi-Square in case of proportion.
Abbreviations are: BMI, body mass index; eGFR, estimated glomerular filtration rate
[1] Including melanomas, excluding all other skin malignancies
* p=0.03
